# Spatial stimulus-response compatibility and affordance effects are not ruled by the same mechanisms

**DOI:** 10.3389/fnhum.2015.00283

**Published:** 2015-05-18

**Authors:** Marianna Ambrosecchia, Barbara F. M. Marino, Luiz G. Gawryszewski, Lucia Riggio

**Affiliations:** ^1^Sezione di Fisiologia, Dipartimento di Neuroscienze, Università di ParmaParma, Italy; ^2^Dipartimento di Psicologia, Università di Milano-BicoccaMilano, Italy; ^3^Neuroscience Program, Universidade Federal FluminenseRio de Janeiro, Brazil

**Keywords:** affordance effect, Simon effect, spatial S–R compatibility, transfer of practice, intact and broken handle

## Abstract

Stimulus position is coded even if it is task-irrelevant, leading to faster response times when the stimulus and the response locations are compatible (spatial Stimulus–Response Compatibility–spatial SRC). Faster responses are also found when the handle of a visual object and the response hand are located on the same side; this is known as affordance effect (AE). Two contrasting accounts for AE have been classically proposed. One is focused on the recruitment of appropriate grasping actions on the object handle, and the other on the asymmetry in the object shape, which in turn would cause a handle-hand correspondence effect (CE). In order to disentangle these two accounts, we investigated the possible transfer of practice in a spatial SRC task executed with a S–R incompatible mapping to a subsequent affordance task in which objects with either their intact handle or a broken one were used. The idea was that using objects with broken handles should prevent the recruitment of motor information relative to object grasping, whereas practice transfer should prevent object asymmetry in driving handle-hand CE. A total of three experiments were carried out. In Experiment 1 participants underwent an affordance task in which common graspable objects with their intact or broken handle were used. In Experiments 2 and 3, the affordance task was preceded by a spatial SRC task in which an incompatible S–R mapping was used. Inter-task delays of 5 or 30 min were employed to assess the duration of transfer effect. In Experiment 2 objects with their intact handle were presented, whereas in Experiment 3 the same objects had their handle broken. Although objects with intact and broken handles elicited a handle-hand CE in Experiment 1, practice transfer from an incompatible spatial SRC to the affordance task was found in Experiment 3 (broken-handle objects), but not in Experiment 2 (intact-handle objects). Overall, this pattern of results indicate that both object asymmetry and the activation of motor information contribute to the generation of the handle-hand CE effect, and that the handle AE cannot be reduced to a SRC effect.

## Introduction

Several studies corroborated the notion that the environment is perceived not only in terms of object visual properties or qualities, but also in terms of object possibilities for action (affordances; Gibson, 1977, 1979/1986). To date, there is ample neurophysiologic evidence demonstrating that the mere observation of common graspable objects recruits the fronto-parietal circuits for object manipulation, both in monkeys (Jeannerod et al., 1995; Murata et al., 1997; Raos et al., 2006; Umilta et al., 2007) and in humans (Grafton et al., 1997; Chao and Martin, 2000; Grèzes et al., 2003; Buccino et al., 2009; Cardellicchio et al., 2011).

At a behavioral level, Tucker and Ellis (1998, 2004), using a Stimulus–Response Compatibility (SRC) paradigm, have presented evidence that visual objects lead to activation of specific components of actions they afford. In a seminal experiment, participants had to judge the vertical orientation (upright or inverted) of common objects with a graspable part (their handle), emitting lateralized key-press responses. Faster reaction times (RTs) were found when the handle and the response key corresponded than when they were on opposite sides, resulting in an affordance effect (AE, Tucker and Ellis, 1998).

Spatial SRC effect (Fitts and Seeger, 1953) refers to faster RTs, in a two-choice key-press task when both the locations of the stimulus and of the response correspond. It happens even if the encoding of the spatial features is not relevant for the response, as in the Simon effect (SE, Simon and Rudell, 1967; Simon, 1969). In a typical Simon task, geometrical shapes are presented on the right or left of the fixation point and participants are instructed to respond to a non-spatial stimulus dimension, such as shape or color, with right or left responses. If, for example, they have to respond with the right response key to green stimuli and with the left response key to red ones, they are faster when green and red stimuli appear on the right and the left side, respectively. As assumed by the dual-process model of the SE (e.g., Kornblum et al., 1990; De Jong et al., 1994), two different response codes would be activated by the stimulus: a code that is automatically activated (primed) by the stimulus spatial location, and a code that is activated by the instructions given to the participants. The corresponding trials lead to more efficient performance because both codes activate the same response. Performance on non-corresponding trials, in contrast, is slower and less accurate because competing responses are activated at the response selection, thus generating a conflict that must be solved before response execution.

It has been suggested that common mechanisms may underlie both SE and AE, given that both effects are based on the spatial relation between the response and the location of the object, or part of it (the handle). Indeed, the object handle could prime responses in its side because of its saliency (e.g., Anderson et al., 2002; Cho and Proctor, 2010, 2011), facilitating the corresponding responses but not the non-corresponding ones, and giving rise to a handle-hand SE. Some authors, indeed, found evidence supporting the hypothesis that AE, far from being the product of the potentiation of appropriate actions to graspable objects, can be the result of an attentional bias toward the handle side of the object. This bias would be produced by the asymmetry of the object, which renders the handle more salient than other object parts (e.g., Anderson et al., 2002; Matheson et al., 2014), thus capturing attention. In keeping with the attention-shift account of the AE (Nicoletti and Umiltà, 1994; Rubichi et al., 1997), this attentional bias would generate a spatial response code, priming the corresponding response (Anderson et al., 2002; Cho and Proctor, 2010; Kostov and Janyan, 2012) as in the typical SE (Proctor and Vu, 2012). However, as showed by Pappas (2014), the results of these studies (e.g., Cho and Proctor, 2010) might be due to the nature of the used stimuli. The author, indeed, compared participants’ performance with naturalistic (photographs) and non-naturalistic stimuli (silhouettes). His findings indicate that the amount of internal details of the objects and the environmental information might be critical to dissociate between SE and AE.

Contrary evidence has also been collected in favor of action-based mechanisms. For example Riggio et al. (2008) reported data supporting the independence between AE and SE. They found that the AE, when evident, was always relative to the target object, irrespective of its attentional capturing properties, whereas the SE occurred relative to the event capturing attention (see also Phillips and Ward, 2002; Symes et al., 2005; Janyan and Slavcheva, 2012). Some studies found that the two effects seem to depend on the stimulus properties being processed in order to perform the task (Pellicano et al., 2010), and that their interaction relies upon the type of action that is required (Iani et al., 2011). Using the same task in which graspable objects were presented, and varying the instructions (i.e., to respond to the color of the object vs. its vertical orientation), either a Simon-like effect or an AE emerged (Pellicano et al., 2010). These two effects interacted in response times but not in reaching movements time (Iani et al., 2011). Furthermore, in a TMS study, Buccino et al. (2009) found that the recruitment of the motor system depends on the graspability of the handle itself, which clearly supports hypotheses based on an action-based role of the handle. In fact when a visual object with a broken handle was presented, that is when the object most important feature relevant for action was violated, motor programs triggered by the handle were violated as well, resulting in significantly reduced MEPs area than when objects were presented with an intact handle.

To investigate whether SE and AE are ruled by common mechanisms, we took advantage of a peculiar feature of SE, that is its susceptibility to the influence of previous practice with an incompatible spatial SRC task (Tagliabue et al., 2000; Vu et al., 2003; Vu, 2007; Creekmur and Vu, 2012). In the transfer of practice paradigm, participants first perform a spatial compatibility task in which they are required to respond with a S–R compatible or incompatible mapping to the stimulus right–left location and then the Simon task. It has been shown that a S–R incompatible mapping eliminates or even reverses the SE (Tagliabue et al., 2000; Vu et al., 2003; Proctor et al., 2007). This demonstrates that the spatial associations between stimulus and response locations defined in the practice task, when stimulus location was relevant, remain active during the subsequent Simon task, when stimulus location is irrelevant. More importantly, this result also shows that in both tasks the same mechanisms are at work since the strategy acquired in the spatial compatibility task (i.e., the strengthening of opposite sides S–R association) transfers to the Simon task.

We explored the possible transfer of practice effect from a spatial SRC task executed with a S–R incompatible mapping to a subsequent affordance task (Tucker and Ellis, 1998). We also manipulated the time between the two tasks (Tagliabue et al., 2000) to assess the possible modulation of time on the duration of the transfer of practice.

Stimuli were common graspable objects with an intact or a broken handle. In the first condition the crucial feature for the expression of AE is preserved, in the second condition it is missing, but the asymmetry of the object and the saliency of the handle are still present. Since only objects with an intact handle should activate specific grasping motor programs, we predicted different practice effects from a spatial SRC task to an affordance task according to the status of the object handle, if the AE and the SE are related to different mechanisms. In order to dissociate between AE and SE we ran three experiments. In Experiment 1 participants had to decide, by pressing a right or a left key, whether pictures depicting common objects, either with an intact or a broken handle, were upright or inverted (affordance task).

In Experiment 1 participants underwent only the affordance task, while in Experiments 2 and 3 the affordance task was preceded by a spatial SRC task executed with a S–R incompatible mapping. In Experiment 2 the objects displayed an intact handle, whereas in Experiment 3 they had a broken handle. In this way we explored the possible practice transfer from an incompatible spatial SRC task to a subsequent intact or broken handle affordance task. If the AE is a Simon-like effect we should expect that it would be nullified or reversed after an incompatible practice as it is for the SE (Proctor and Lu, 1999; Tagliabue et al., 2000; Vu et al., 2003; Vu, 2007) either when the handle is intact or broken. In contrast, if AE and SE are the result of different mechanisms then we should not expect transfer of practice from a spatial SRC to an affordance task, at least when objects with the intact handle are used. The inter-task delay was manipulated in both experiments (5 vs. 30 min).

## Materials and Methods

### Experiment 1

Experiment 1 was designed to test if the handle-hand correspondence effect (CE) occurs relative to the object’s handle, and if its magnitude depends on the graspability of the handle. To this aim, participants were required to respond to upright or inverted graspable objects presented at the center of a computer screen. Objects could have their handle intact or broken.

#### Participants

Twenty-four undergraduate students (16 females; mean age = 22.5 ± 4.5) from the University of Parma volunteered to take part in this experiment. All participants were right-handed as assessed by the Edinburgh Handedness Inventory (Oldfield, 1971), had normal or corrected-to-normal vision and were naïve as to the purpose of the experiment. The experimental protocol was approved by the Ethics Committee of the University of Parma. The experiment was conducted in accordance with the ethical standards of the 1964 Declaration of Helsinki and all participants gave written informed consent.

#### Procedure

The experiment was carried out in a sound-attenuated room, dimly illuminated by a halogen lamp directed toward the ceiling. The participants were tested individually. They sat comfortably in front of a computer screen (Philips monitor with a resolution of 1024 × 768 pixels, interfaced with a Pentium 2.80 GHz computer equipped with a NVIDIA GeForce 7300 LE video Board), mounted in a wooden frame and covered by a gray cardboard, except for a 18 cm × 25.5 cm window where the stimuli were displayed. Participants had their head supported by a chin rest in order to maintain a stable head position and keep their eyes at a constant distance from the screen (about 57 cm). Eye height was adjusted to the level of fixation.

**FIGURE 1:**
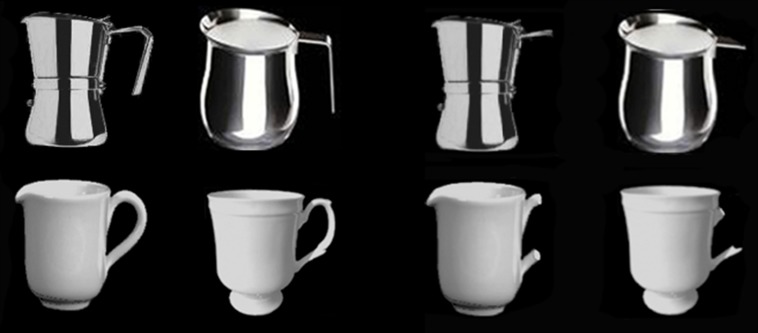
**Stimuli used in the affordance task**.

Stimuli presentation and response collection were controlled by E-Prime software system. The stimuli consisted of a series of images of four common objects (a coffee pot, a milk jug, a tea-cup, and a coffee cup; see **Figure 1**) presented in two vertical orientations (upward or inverted). Each object was inserted in a 157 × 126 pixels matrix and was displayed in gray scale on a black background at the center of the screen. All objects had a handle, intact or broken, oriented to the right or the left (suitable for a right-hand or a left-hand grasp).

Responses were executed by pressing the “P” or the “Q” key of the QWERTY keyboard with the left and the right index finger, respectively. The response keys were in symmetrical locations to the right and the left of the body midline. Participants were requested to keep their index fingers on the keys during the experiment.

Each trial began with the presentation of the fixation cross (22 × 22 pixels), replaced after 500 ms by an upward or inverted object in the center of the screen, with its handle oriented to the right or to the left. Twelve participants executed the task with intact handled objects and twelve with broken handled objects. Objects were displayed until a response was given; if the response did not occur within 1000 ms, the object disappeared. Half of the participants were instructed to make a left key-press (Q) for upright objects and a right key-press (P) for inverted objects. The remaining participants experienced the reverse mapping. Visual feedback on speed and accuracy was provided for 500 ms in the center of the screen after each response.

The experiment consisted of 160 experimental trials divided into two blocks of 80 trials each. The first block was preceded by sixteen familiarization trials with the same stimuli used in the experimental trials. For each handle condition (intact or broken) an equal number of trials was provided for each combination of the following variables: object Orientation (upward vs. inverted) and handle-hand Correspondence (corresponding vs. non-corresponding).

The correct mean response latencies (RTs) and accuracies (following arcsine transformations) were entered into two analyses of variance (ANOVAs), with object Orientation (up vs. down) and hand-handle Correspondence (corresponding vs. non-corresponding) as within-subjects variables, and Handle (intact vs. broken) as a between-subjects variable. Whenever appropriate, *post hoc* analyses were conducted using the Tukey’s HSD (honest significant difference) test in order to control for both the Type I and Type II errors, since it is not only more conservative (e.g., the Newman–Keuls method), but also more powerful than other procedures (e.g., the Bonferroni method; Perneger, 1998).

#### Results and Discussion

Familiarization trials were discarded from the analysis. Overall errors (wrong responses and missing responses) were 7.4% of the dataset. Responses either longer or shorter than two SDs from the individual mean were treated as outliers and not considered in the analysis (2% of the data set).

The ANOVA on Accuracy revealed that only the main effect of Correspondence was significant (*F*_1_,_22_ = 15.81; *p* < 0.01; η^2^ = 0.42), indicating that participants were more accurate for corresponding (mean = 95%, SE = 1.43) than for non-corresponding trials (mean = 90.16%, SE = 1.42)

**FIGURE 2:**
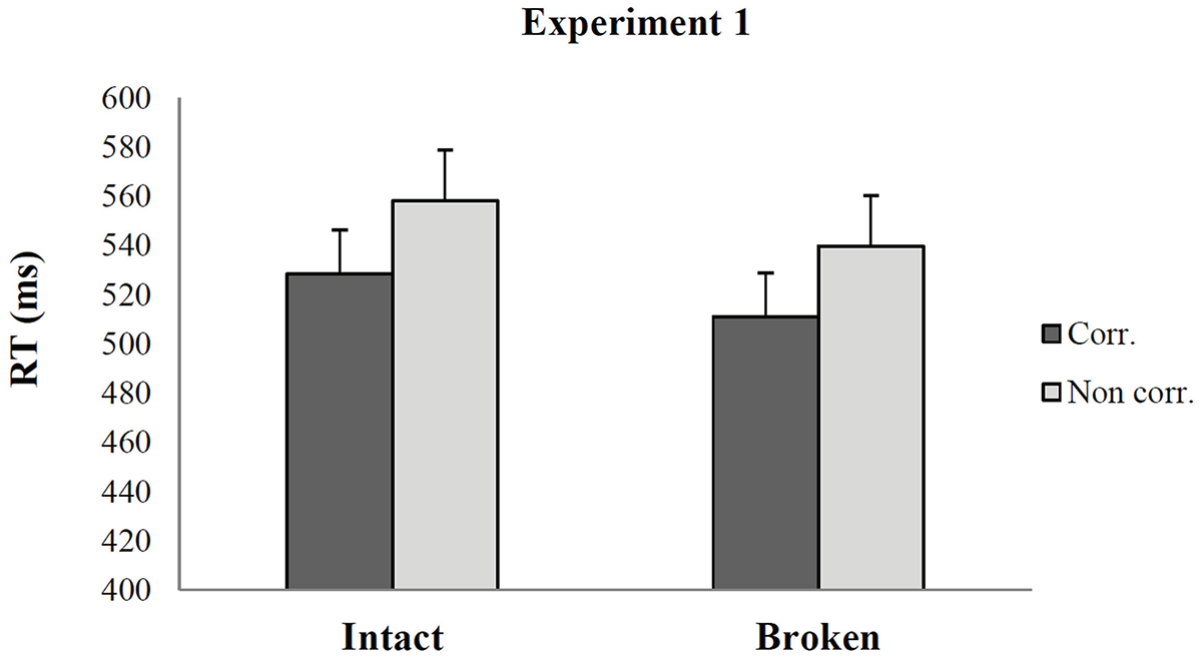
**Mean RTs in Experiment 1 for corresponding and non-corresponding trials as a function of the status of the object handle**. The main effect of Correspondence is significant, but not the interaction between Correspondence and Handle. Error bars depict SEs of the means.

Similarly, the ANOVA on RTs revealed that only the main effect of Correspondence was significant (*F*_1_,_22_ = 43.91; *p* < 0.001; η^2^ = 0.66). Importantly both the analysis on Accuracy and the analysis on RTs showed no real difference on overall responses between objects that had an intact or a broken handle pointing out that they were equally recognizable. The interaction between Correspondence and Handle was not significant (*F*_1_,_22_ = 0.012; *p* > 0.9; η^2^ = 0.001) demonstrating that the magnitude of the handle-hand CE when the handle was either intact or broken is very similar [Δ RTs (non-corresponding – corresponding) = 29.7 ms, SE = 4.4 vs. 28.7 ms, SE = 7.6; see **Figure 2**].

As evidenced by a large number of studies, Experiment 1 confirms that when objects with intact handles oriented to the right or to the left are displayed, responses are faster if the location of the response corresponds to the location of the handle. Notably, the same effect is also obtained when objects with a broken handle are shown. Since in this case the handle does not afford grasping, the handle-hand CE cannot be regarded as an AE, suggesting that it might be produced by the asymmetry of the object more than the pragmatic role of the handle. Given the similarity of results when objects with an intact or broken handle are presented, a parsimonious explanation might refer both effects to an attentional bias toward the asymmetrical part of the object, i.e., the handle intact or broken. Matheson et al. (2014) reached a similar conclusion in a study in which asymmetrical manipulable artifacts and non-manipulable animals were compared in a SRC paradigm. With both types of stimuli, compatibility effects were reported. However, as the same authors argued, the fact that compatibility effects were obtained both with artifacts and animals does not exclude *per se* that different mechanisms could be at work. Therefore, on the basis of studies showing that the AE is not merely a kind of SE (Symes et al., 2005; Riggio et al., 2008; Buccino et al., 2009; Pappas, 2014), we think that the similarity between the two handle-hand CE, found in the present study, is only apparent. Thus solely the observed handle-hand CE when objects with their intact handle were displayed might be due to the recruitment of handle grasping information. The study of Buccino et al. (2009), which to our knowledge is the unique study that used objects with intact and broken handles, seems to support this interpretation. In fact, it clearly demonstrated that the status of the handle is critical in the recruitment of the motor system. Hence Experiments 2 and 3 specifically try to dissociate the handle-hand CE due to the intact and the broken handle.

### Experiment 2

In order to investigate whether SE and AE depend on the same mechanisms, in this experiment we explored the possible practice transfer from a spatial SRC task executed with a S–R incompatible mapping to a subsequent affordance task with objects having their intact handle (Tucker and Ellis, 1998). The time between the two tasks has been manipulated (5 vs. 30 min).

#### Participants

Twenty-eight undergraduate students (12 females; mean age 21.66 ± 3.71) volunteered to take part in this experiment. None of them took part in the previous experiment. They were right-handed, had normal or corrected-to-normal vision and were naïve as to the purpose of the experiment. All participants gave written informed consent. Experiments were conducted in accordance with the ethical standards of the 1964 Declaration of Helsinki.

#### Procedure

In this experiment participants had to perform two successive tasks: a spatial SRC task with an incompatible S–R mapping and, after a delay of 5 or 30 min, an affordance task. Fourteen participants executed the affordance task with objects having their intact handle with the inter-task interval of 5 min and fourteen executed the task with the inter-task interval of 30 min.

#### The Spatial SRC Task

Each trial began with the presentation of a central fixation cross (22 × 22 pixels), followed after 500 ms by a white circle (30 × 30 pixels) presented 10∘ (center to center) to the right or to the left of the fixation. The circle remained until a response was given, but anyway no longer than 1000 ms.

Participants were instructed to respond to the location (left or right) of the circle by pressing the key on the opposite side, that is the left response key (Q) when the circle compared to the right and the right response key (P) when it compared to the left. Visual feedback on speed and accuracy was provided after response was given.

The task was composed by 160 experimental trials divided into two blocks of 80 trials each. Sixteen familiarization trials preceded the first block. An equal number of trials was provided for the left and right stimulus location.

A two-tailed independent samples *t*-test was carried out both on mean RTs for correct responses and accuracies (following arcsine transformations), comparing the performance in the practice task between the two groups of participants (participants who executed the affordance task after 5 or 30 min the practice task).

#### The Intact Handle Affordance Task

Stimuli and procedure of the affordance task were the same as in the Experiment 1. Mean RTs for correct responses and accuracies (following arcsine transformations) were submitted to two ANOVAs with Orientation (up vs. down) and handle-hand Correspondence (corresponding vs. non-corresponding) as within-subjects variables, and inter-tasks Time (5 vs. 30 min) as a between-subjects variable. A further ANOVA was conducted on Δ RTs comparing the magnitude of the handle-hand CE among the three practice conditions used in Experiments 1 and 2. The handle-hand CE was entered in a one-way ANOVA with Practice (absent vs. 5 min before the task vs. 30 min before the task) as a between-subject variable. *Post hoc* analyses were conducted using the Tukey’s HSD method.

#### Results and Discussion

##### Results of the Spatial SRC Task

Familiarization trials were discarded from the analysis. Errors (wrong responses about the position of the circle and missing responses) were 1% of the total trials. Responses either longer or shorter than two SDs from the individual mean were treated as outliers and not considered (3.8% of the data set).

Both *t*-test comparisons on accuracy (*t*_26_ = 0.07, *p* > 0.9; 2-tailed) and RTs (*t*_26_ = -1.43, *p* > 0.1; 2-tailed) showed that the performance in the practice task did not differ between the group of participants assigned to the 5 min condition (mean RTs = 388 ms, SE = 10; mean accuracy = 98%, SE = 1) and the group assigned to the 30 min condition (mean RTs = 417 ms, SE = 4; mean accuracy = 98%, SE = 0.5).

##### Results of the Intact Handle Affordance Task

Familiarization trials were discarded from the analysis. Errors (wrong responses about orientation of the object and missing responses) were 5% of the total trials. Responses either longer or shorter than 2 SDs from the individual mean were treated as outliers and not considered (0.3% of the data set).

The ANOVA on Accuracy showed that only the main effect of Correspondence was significant (*F*_1_,_26_ = 23.17; *p* < 0.01; η^2^ = 0.47), with more accurate responses in corresponding trials (mean = 96%, SE = 1.84) than in non-corresponding ones (mean = 90%, SE = 1.98).

**FIGURE 3:**
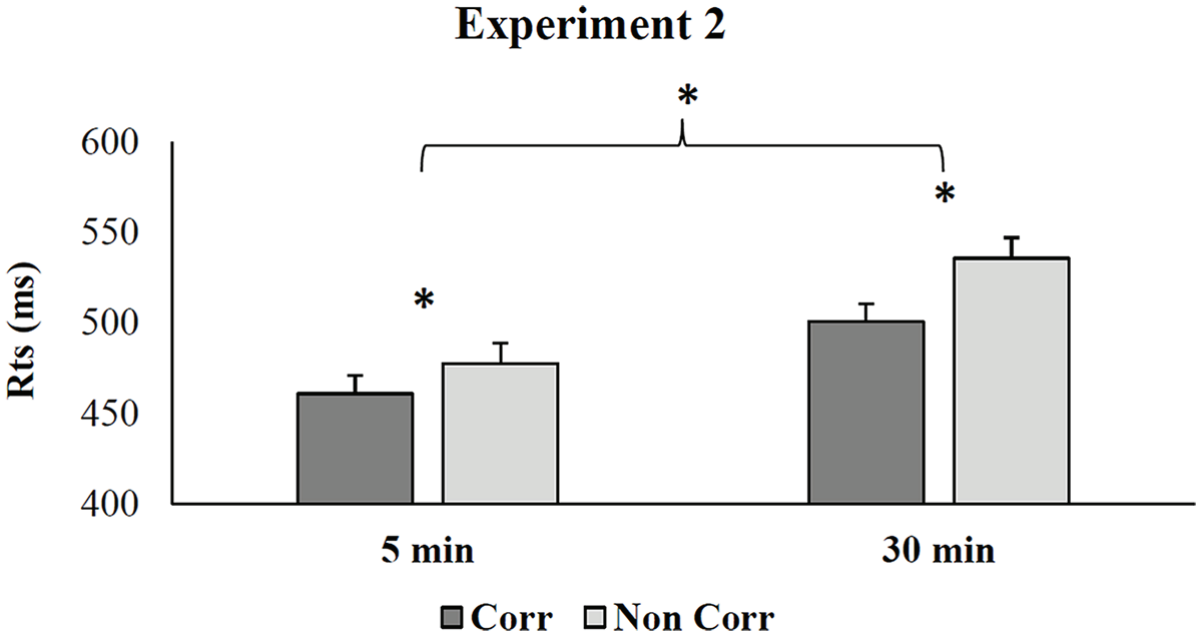
**Mean RTs during the affordance task in Experiment 2 for corresponding and non-corresponding trials as a function of inter-tasks time**. Error bars depict SEs of the means. The asterisk indicates a statistical significance between the means.

The ANOVA on RTs revealed that the main effect of Time was significant (*F*_1_,_26_ = 10.99; *p* < 0.01; η^2^ = 0.30) showing that responses were faster (mean = 469 ms, SE = 10.4) when the two tasks were separated by 5 min than by 30 min (mean = 518 ms, SE = 10.4). Also the main effect of Correspondence was significant (*F*_1_,_26_ = 50.08; *p* < 0.01; η^2^ = 0.61) with faster RTs in corresponding (mean = 481 ms, SE = 7.10) than non-corresponding trials (mean = 506 ms, SE = 8). Moreover Correspondence interacted significantly with Time (*F*_1_,_26_ = 6.55; *p* < 0.05; η^2^ = 0.2). *Post hoc* comparisons revealed a handle-hand CE of a smaller magnitude in the shorter than in the longer inter-tasks condition (Δ = 16 ms, SE = 4 vs. 35 ms, SE = 6; *p* < 0.03 see **Figure 3**).

The ANOVA on Δ RTs showed that the main effect of Practice was significant (*F*_2_,_37_ = 3.88; *p* < 0.03; η^2^ = 0.2). The magnitude of the handle-hand CE in the 5 min delay before the Affordance task was smaller than the 30 min delay condition (mean = 16, SE = 4.85 vs. mean = 35 ms, SE = 4.85; *p* < 0.03) and largely reduced than the no practice condition (mean = 16 ms, SE = 4.85 vs. mean = 30 ms, SE = 5.24; *p* = 0.16) even if the difference was not significant. Moreover, the magnitude of the handle-hand CE in the 30 min delay condition did not differ significantly from the no practice condition (mean = 35 ms, SE = 4.85 vs. mean = 30 ms, SE = 5.24; *p* > 0.4).

**FIGURE 4:**
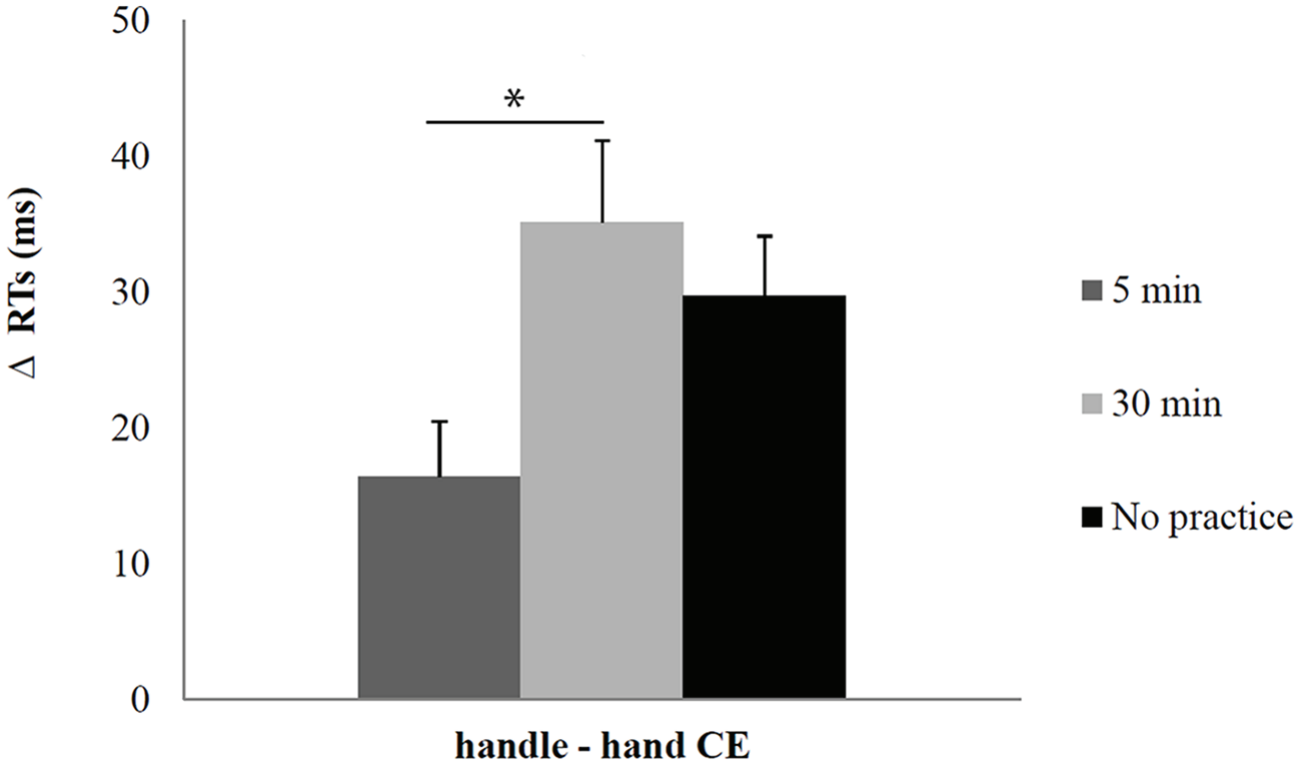
**Handle-hand CE in Experiments 1 and 2 during the affordance task**. Error bars depict SEs of the means. The asterisk indicates a statistical significance between the means.

The data demonstrated that the handle-hand CE is modulated by previous practice with a smaller magnitude in the short but not in the long inter-tasks time condition (see **Figure 4**).

Although, our results may be connected to a briefer effect of incompatible practice on AE than on SE, previous studies have found that the magnitude of the handle-hand CE depends on the response speed (see Supplementary Material), with an increasing magnitude over time (Phillips and Ward, 2002; Fischer and Dahl, 2007; Proctor et al., 2011). This means that participants would exhibit a handle-hand CE smaller in fast than in slow RTs. Since in this experiment participants’ performance was significantly faster in the 5 min than in the 30 min time condition, it follows that the reduction of the magnitude of the handle-hand CE in the 5 min condition may be due to response speed rather than to a short transfer effect of practice on AE. To disentangle the role of practice and response speed, following the Vincentization procedure introduced by Ratcliff (Ratcliff, 1979), we divided the RT distributions for each participant, and for the two levels of Time variable, into four quantiles (bins) and we computed mean RTs for each quantile (for further analyses on Experiments 1 and 2, see Supplementary Material).

**FIGURE 5:**
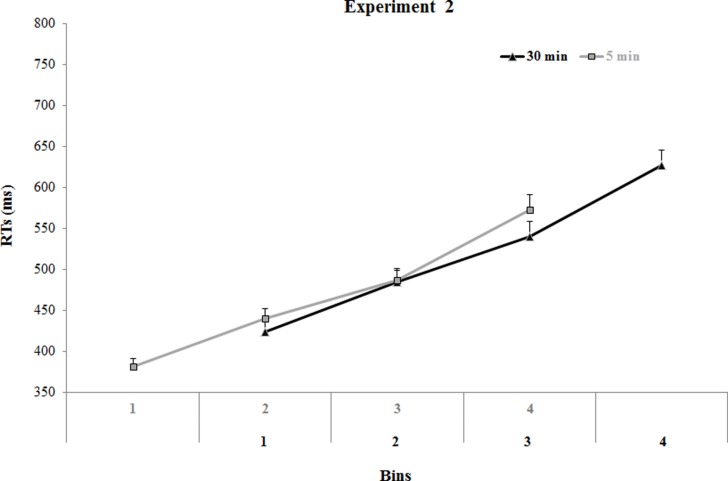
**Overall mean RTs in Experiment 2 as a function of the four bins and the two inter-tasks time**. The first three bins of the 30 min condition (in black) overlap with the last three bins of the 5 min condition (in gray). Error bars depict SEs of the means.

This RT distribution analysis indicated that in order to eliminate the effect due to differences in response speed, it was necessary to compare only overlapping bins: the first three bins of the 30 min condition with the last three bins of the 5 min condition. To make this kind of comparisons we adopted three two-tailed independent samples *t*-test (see **Figure 5**; all *p*s > 0.10). Based on the results of these analyses, mean data for the second, third, and fourth bins of the 5 min condition and the first, second, and third bins of the 30 min condition were entered in the ANOVA. Bin and Correspondence were within-subjects variables, and Time (5 vs. 30 min) was a between-subjects variable. As before, when necessary, *post hoc* comparisons were performed using the Tukey’s HSD method.

Besides the main effect of Bin, (*F*_2_,_52_ = 801.90, *p* < 0.001; η^2^ = 0.97), the analysis revealed the main effect of Correspondence (*F*_1_,_26_ = 52.72, *p* < 0.01; η^2^ = 0.68). The interaction between Bin and Correspondence was also significant (*F*_2_,_52_ = 5.22, *p* < 0.005; η^2^ = 0.17), showing that the magnitude of the handle-hand CE increases as reaction times become slower (Bin 1: Δ = 19.27 ms; Bin 2: Δ = 27.87 ms; Bin 3: Δ = 32.3 ms; all *p*s < 0.001), as typically shown for the AE (Phillips and Ward, 2002; Fischer and Dahl, 2007; Proctor et al., 2011). However, the interaction among Bin, Correspondence and Time was not significant (*F*_12_,_52_ = 0.72, *p* > 0.5; η^2^ = 0.27), revealing that the reduction of the magnitude of the hand-handle CE in the 5 min was due to the response speed rather than to the previous practice in incompatible spatial SRC. Hence, although one possible interpretation of the larger handle-hand CE at 30 min is that the interference of the prior practice has worn off by then in this task, our results support, instead, the absence of a transfer effect of the incompatible practice on the AE.

### Experiment 3

Experiment 2 showed no transfer effect from an incompatible SRC task to a subsequent intact Handle Affordance Task supporting different acting mechanisms in the two tasks. Experiment 3 was set to assess the transfer effect when objects having a broken handle are presented in the affordance task.

#### Participants

Twenty-eight undergraduate students (19 females; mean age 20.82 ± 4.26) volunteered to take part in this experiment. All students were right-handed, had normal or corrected-to-normal vision and were naïve as to the purpose of the experiment. All participants gave written informed consent. Experiments were conducted in accordance with the ethical standards of the 1964 Declaration of Helsinki.

#### Procedure

As in Experiment 2, participants had to perform two tasks: the incompatible spatial SRC task and, after a delay of 5 or 30 min, the affordance task. However, in this case all the objects were presented with the broken handle. Fourteen participants executed the task with the inter-task interval of 5 min and 14 executed the task with the inter-task interval of 30 min.

For the practice task, a two-tailed independent samples *t*-test was carried out both on mean RTs for correct responses and accuracies (following arcsine transformations) comparing the performance to the practice task between the two group of participants (participants who executed the affordance task after 5 or 30 min the practice task).

Regarding the Broken Handle Affordance Task, correct responses (Accuracy) and mean RTs for correct responses were submitted to an ANOVA with Orientation (up vs. down) and Correspondence (corresponding vs. non-corresponding) as within-subjects variables, and inter-tasks Time (5 min vs. 30 min) as a between-subjects variable. A further ANOVA was conducted on ΔRTs comparing the magnitude of the handle-hand CE in the two inter-task Time (5 min vs. 30 min) conditions. As before *post hoc* comparisons were performed using the Tukey’s HSD method.

#### Results and Discussion

##### Results of the Spatial SRC Task

Familiarization trials were discarded from the analysis. Errors (wrong responses about the position of the circle and missing responses) were 1% of the total trials. Responses either longer or shorter than 2 SDs from the individual mean were treated as outliers and not considered (3.9% of the data set).

Both *t*-test comparisons on accuracy (*t*_26_ = 0.65, *p* > 0.5; 2-tailed) and RTs (*t*_26_ = 1.65, *p* > 0.1; 2-tailed) showed that the performance in the practice task did not differ between the group of participants assigned to the 5 min condition (mean RTs = 419 ms, SE = 16; mean accuracy = 98%; SE = 0.5) and the group assigned to the 30 min condition (mean RTs = 380 ms, SE = 16; mean accuracy = 98%; SE = 0.5).

##### Results of the Broken Handle Affordance Task

Familiarization trials were discarded from the analysis. Overall errors (wrong responses about orientation of the object and missing responses) were 5.6% of the total trials. Responses either longer or shorter than 2 SDs of the individual mean were treated as outliers and not considered (0.6% of the data set).

**FIGURE 6:**
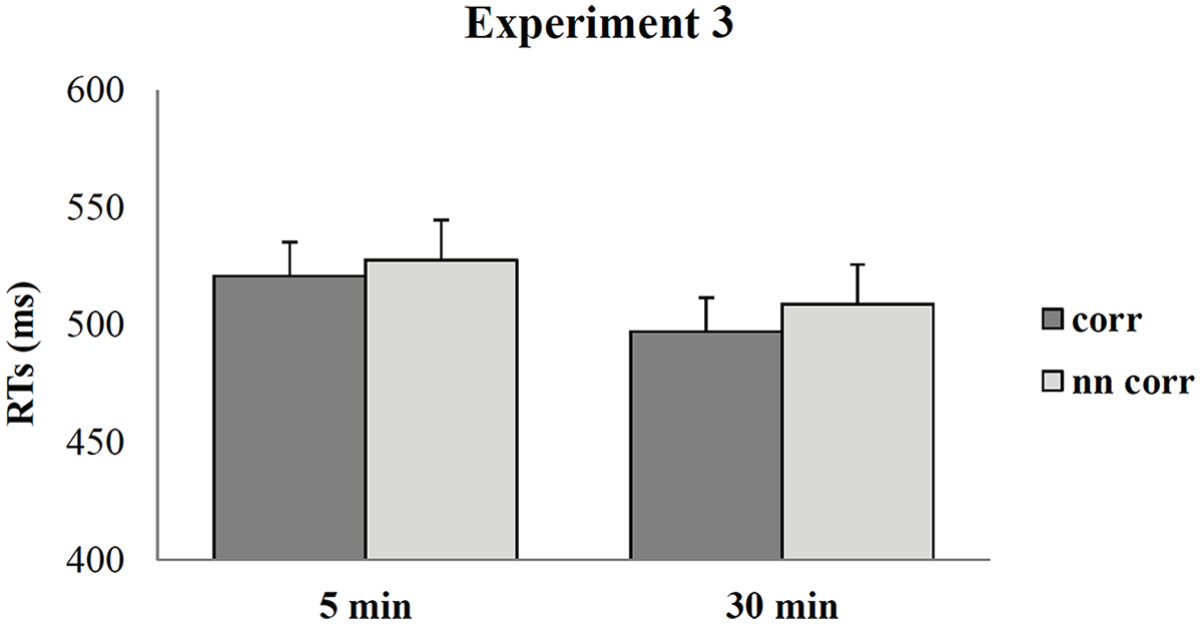
**Mean RTs during the affordance task in Experiment 3 as a function of correspondence and time**. Error bars depict SEs of the means.

Both the analyses on Accuracy and RTs did not reveal any significant effect or interaction. In particular, neither the handle-hand Correspondence (*F*_1_,_26_ = 1.37; *p* = 0.064; η^2^ = 0.05), nor the interaction between handle-hand Correspondence and Time (*F*_1_,_26_ = 0.25; *p* = 0.61; η^2^ = 0.01) were significant in the RT analysis, although corresponding trials were slightly faster than non-corresponding ones (512 ms, SE = 11.07 vs. 518 ms, SE = 11.21; see **Figure 6**).

### Experiment 2 vs. Experiment 3

In order to compare the handle-hand CE in Experiments 2 and 3, we ran a bin distributional analysis, computing correct means RTs from the first to the fourth bin of the individual rank-ordered raw data separately for the Handle Status (intact vs. broken) and Time (5 min vs. 30 min) variables using the same procedure adopted in the Experiment 2.

**FIGURE 7:**
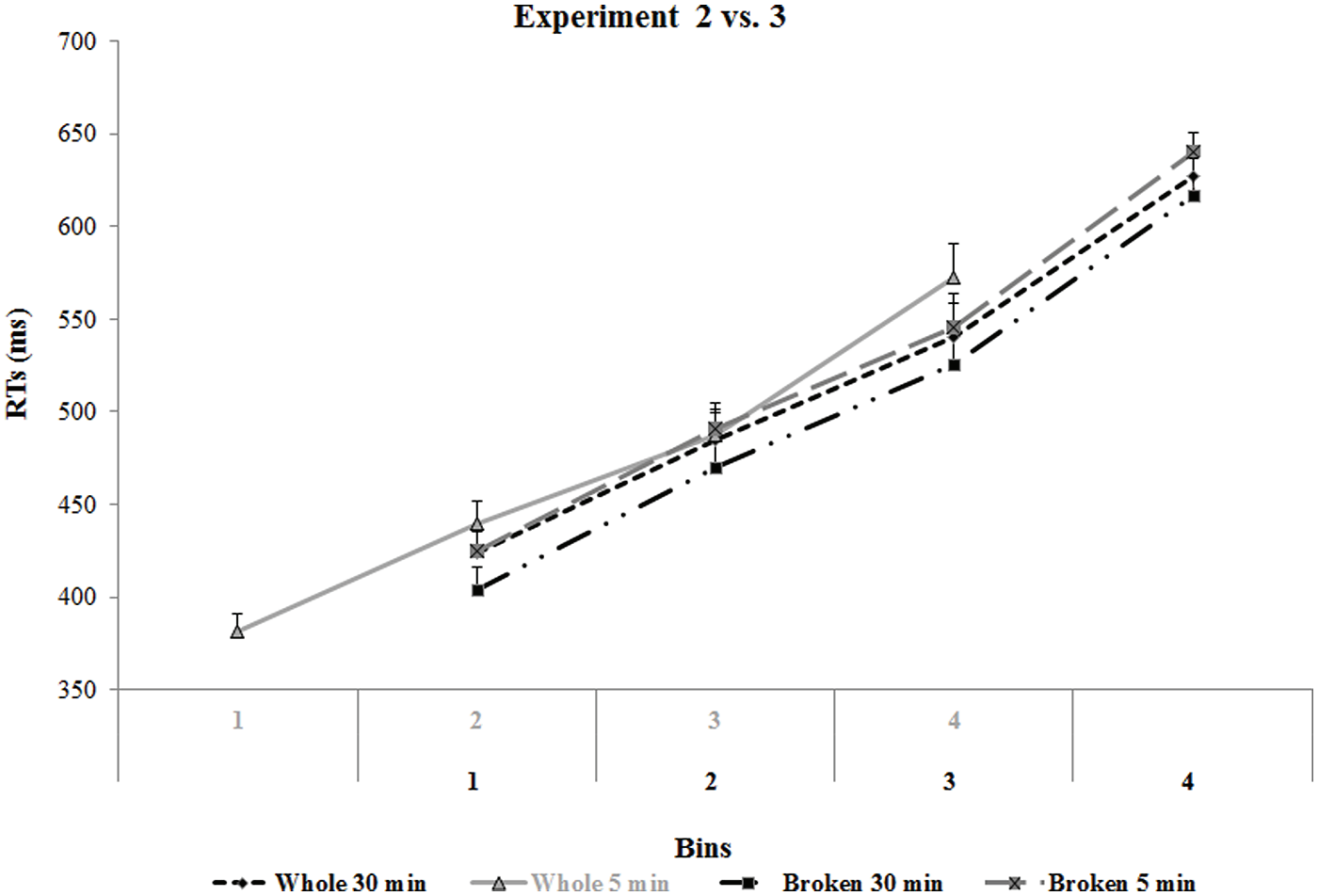
**Mean RTs in the affordance task in Experiments 2 and 3 as a function of bin, time, and handle**. In Experiment 1 (intact handle), the last three bins of the 5 min condition (in gray) overlap with the first three bins of the 30 min condition, and the last three bins of the 5 and 30 min condition of Experiment 3 (broken handle; in black). Error bars depict SEs of the means.

Mean data were entered into an ANOVA in which we considered only overlapping bins (see **Figure 7**; all *p*s > 0.05; pairwise *t*-test comparisons). Correspondence (corresponding vs. non-corresponding) and Bin were within-subjects variables and Handle Type (Intact vs. Broken) and Inter-task Time (5 vs. 30 min) were between-subjects variables. As before, when necessary, *post hoc* comparisons were performed using the Tukey’s HSD method.

**FIGURE 8:**
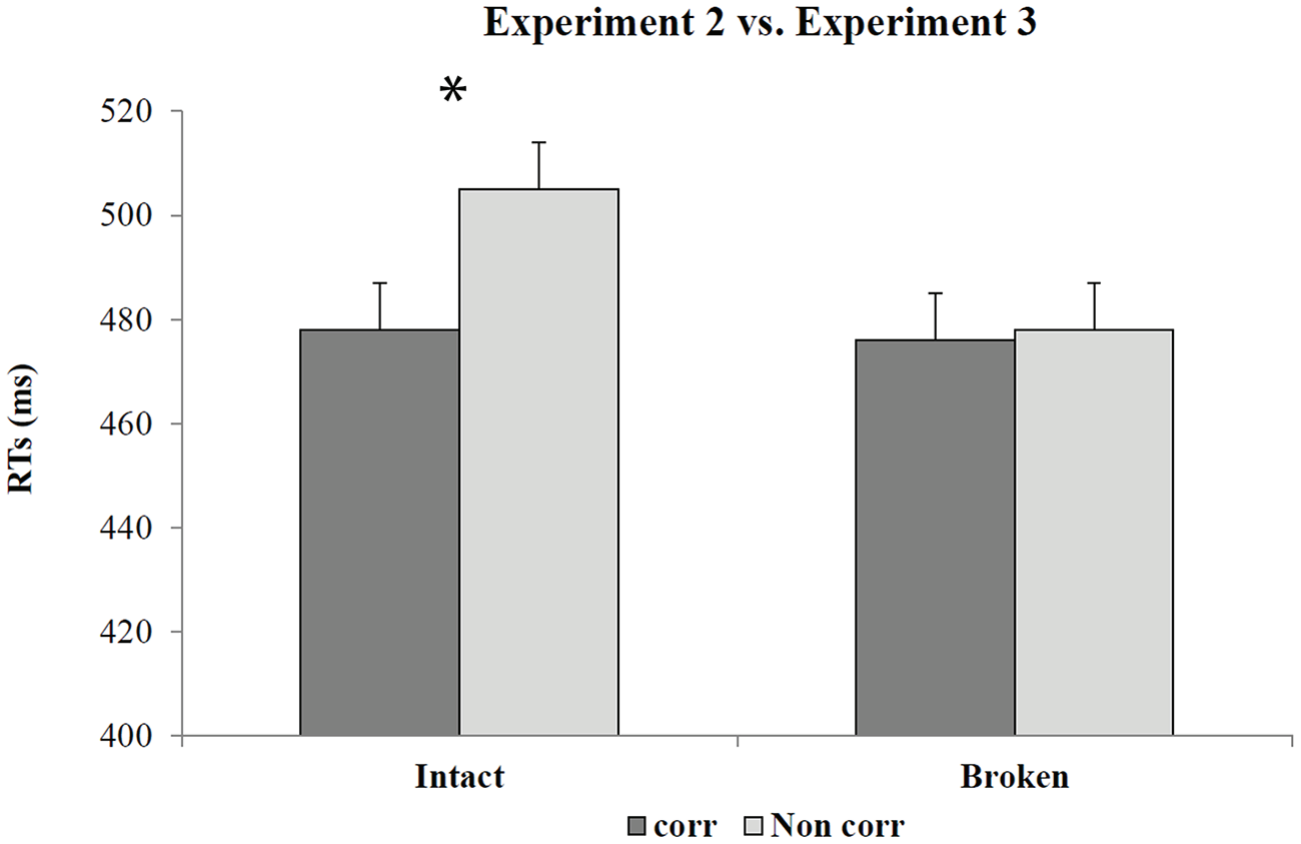
**Mean RTs as a function of correspondence and handle status during the affordance task in Experiments 2 and 3 comparing similar bins**. Error bars depict SEs of the means. The asterisk indicates a statistical significance between the means.

Besides the main effect of Bin, (*F*_2_,_104_ = 1220.40; *p* < 0.001; η^2^ = 0.96), the analysis revealed the main effect of Correspondence (*F*_1_,_52_ = 25.02; *p* < 0.01; η^2^ = 0.33). The interaction between Correspondence and Handle was also significant (*F*_1,52_ = 17.97; *p* < 0.01; η^2^ = 0.26). *Post hoc* comparisons showed that after an incompatible spatial SRC practice, the handle-hand CE occurs only in the intact handle affordance task (intact handle affordance task = 478 ms, SE = 9 vs. 505 ms, SE = 9, *p* < 0.001; broken handle affordance task = 476 ms, SE = 9 vs. 478 ms, SE = 9, *p* = 0.5; for corresponding and non-corresponding trials, respectively; see **Figure 8**).

## General Discussion

The present study aimed at disentangling between the two main accounts of the handle-hand CE: the recruitment of motor programs for interacting with the object and the orienting of attention toward the asymmetrical part of the object. To this end, we assessed the possible transfer of practice from a prior incompatible spatial SRC task to an affordance task in which objects, with an intact or broken handle, were presented. Indeed, while the presence of the intact handle makes the recruitment of motor programs for handle grasping possible, a broken handle prevents it.

Results of the Experiment 1 showed a handle-hand CE of the same magnitude regardless of the handle status (30 vs. 29 ms for the intact and broken handle, respectively). This result may support the orienting attention hypothesis (e.g., Anderson et al., 2002; Cho and Proctor, 2010), and hence the fact that the AE could be simply reduced to a SE, due to the asymmetry of the object; or, in other words, to the spatial relation between the location of the handle, either when it is intact or broken, and the location of the response. If this was true, and hence the two effects would be based on the same mechanisms, we should expect that both of them would be nullified or reversed after an incompatible practice (Proctor and Lu, 1999; Tagliabue et al., 2000; Vu et al., 2003; Vu, 2007).

This is what we observed in Experiment 3, where objects with their broken handle were presented. The handle-hand CE after the incompatible practice was eliminated with an inter-task interval of both 5 and 30 min (no practice = 29; 5 min = 5; 30 min = 6 ms). In contrast, in Experiment 2, in which objects with their intact handle were used, no real transfer effect was found. In fact, although the handle-hand CE apparently diminished after a delay of 5 min in comparison to both the 30 min delay and the task executed without prior practice (no practice = 30; 5 min = 16; 30 min = 35 ms), it may be that this reduction depends on different response speed between the two delay time conditions. It is well known that AE increases as the RTs increase (Phillips and Ward, 2002; Fischer and Dahl, 2007; Proctor et al., 2011). Thus, to assess the modulation of the prior practice on the subsequent affordance task, it is important to consider the response speed by comparing similar RTs. After such a RT adjustment, the handle-hand CE seems not to be influenced by the prior incompatible practice when objects having their intact handle were presented, in agreement with an AE account. Conversely, the handle-hand CE was eliminated by the prior incompatible practice when objects with their handle broken were shown, as was expected for a SE. These results suggest that the AE and SE may be considered independent.

However, since the handle makes objects asymmetric, determining a bias for attentional shift, it is reasonable to wonder whether the handle-hand CE comprises also a SE, as it could be deduced from the presence of the handle-hand CE in Experiment 1 with objects with a broken handle. In this condition indeed, the broken handle, because of its non-graspability, does not trigger manual motor programs. There are more than one reason against this idea: the magnitude of the handle-hand CE observed in Experiment 1 does not differ between intact and broken handle objects and, when the handle is intact and graspable, the practice does not modulate it. On the contrary the handle-hand CE becomes null when the handle is broken suggesting the presence of a SE. Therefore, the two effects (AE and SE) are not additional but seem to compete.

These results are in line with Symes et al. (2005) and Riggio et al. (2008). In the first study the authors by manipulating the orientation and the location of the objects found two separate SRC effects at different levels of attentional demand; the first one was a SE, which appeared alone when the attentional demand was low; the second one, due to the orientation of the handle, required that the object was coded as an object. In the second study, the event capturing attention was manipulated to assess the role of attention in the emergence of AE and SE. The authors found that the AE, when evident, was always relative to the target object, irrespective of its attentional capturing properties; while the SE was present in relation to the event capturing attention. A recent study by Wilf et al. (2013) gave additional evidence of the independence between the SE and AE. These authors, by measuring button-press and electromyography (EMG) responses, found the presence of spatial SRC from the earliest stages of movement preparation and throughout the different stages of movement execution. In contrast, the AE was evident only in the early stages of movement execution, although this effect has been only related to a general motor system activation, and not specifically connected to a body-part. They tested also a small group of unilateral amputees using EMG and found residual spatial SRC but no AE.

Unlike our results, a recent study by Ottoboni et al. (2013) found that an incompatible practice eliminated the AE. Although these authors did not aim at directly dissociate between SE and AE, they interpreted this result as an evidence supporting that AE and SE share some similarities (Iani et al., 2011; McBride et al., 2012). A reason for the difference between their and our study could regard the different amount of practice in the two studies (160 vs. 600 trials in the present and in Ottoboni et al.’ (2013) study, respectively).

Several studies (e.g.,Tagliabue et al., 2000; Vu et al., 2003; Vu, 2007) showed that the transfer from a spatially incompatible practice to a subsequent Simon task is already evident after 72 practice trials and it lasts up to 1 week (Tagliabue et al., 2000). Vu (2007) indicated, however, that while a short practice (e.g., 72 trials) could be sufficient to give rise to a “within-dimension transfer effect” (e.g., from an incompatible horizontal SRC practice to a subsequent horizontal Simon task), a “between-dimension transfer effect” (e.g., from an incompatible horizontal SRC practice to a vertical Simon task) needs up to 600 trials of incompatible practice to emerge. Some authors (Wiegand and Wascher, 2005; Vu, 2007) suggested that the within-dimension transfer effect may be due to the short-term spatial S–R associations, acquired with short practice, overriding the long term associations, and the between-dimension transfer effect may be due to the acquisition of a more general strategy of giving a response opposite to stimulus location (Vu, 2007). Also, Marini et al. (2011) reported that after 600 trials, practice transfer is present even if the two tasks do not share any spatial irrelevant dimension. They found a significant reduction of the subsequent color Stroop effect (Stroop, 1935; see MacLeod, 1991 for a review) after an incompatible spatial SRC practice. However, this transfer effect was absent if the subsequent Stroop task did not require the same response modality of the practice task (i.e., vocal responses instead of bimanual responses). These findings demonstrated that in order for the transfer effect to appear, rather than the dimensional overlap between stimuli and responses of the two tasks, it needs the dimensional overlap between the responses of the two tasks. It seems that after 600 trials of practice participants learned to emit the response alternative to the one automatically activated and that such a rule transferred into the following task. This cognitive strategy could be also responsible of the elimination of the AE found by Ottoboni et al. (2013). Although Ottoboni et al. (2013) demonstrated that, as for the SE, the conflict at the basis of the AE is not unavoidable, this is only an indirect index of the mechanisms at the basis of the two effects. These results alone, indeed, do not allow us to disentangle between the two accounts of the handle-hand CE.

In the present study, using the same amount of practice, we compared the transfer effect between objects having their intact or broken handle. As described above, such an amount of practice is enough to eliminate the SE. If it was true that the AE is a SE, we should have found the elimination of the handle-hand CE both in Experiments 2 and 3. Furthermore, the transfer effect should have remained even after 30 min.

The fact that the handle-hand CE for objects with their intact graspable part was not influenced by the prior practice, unlike what happens for objects with their broken handle, is compelling evidence that the two observed effects are due, at least in part, to different mechanisms.

## Conclusion

Our findings support the motor-based nature of handle-hand CE in spatial SRC paradigms. We found that both the activation of motor programs and the asymmetry of the object because of the handle, can contribute to the generation of the handle-hand CE, but while the former leads to the generation of the AE, the latter leads to the generation of a Simon like CE when the handle is not graspable because it is broken. Hence the graspable part of an object is a condition not necessary to generate a SE, but it is necessary to generate an AE.

## Conflict of Interest Statement

The authors declare that the research was conducted in the absence of any commercial or financial relationships that could be construed as a potential conflict of interest.
