# Supplementary material for: Spatial stimulus-response compatibility and affordance effects are not ruled by the same mechanisms
Source: Front Hum Neurosci. 2015 May 18;9:283. doi: 10.3389/fnhum.2015.00283 (PMC4435042; doi:10.3389/fnhum.2015.00283)
Supplement: Supplementary file 1 [file Data_Sheet_1.PDF]

# *Supplementary Material*

# Spatial stimulus-response compatibility and affordance effects are not ruled by the same mechanisms

Marianna Ambrosecchia[1], Barbara F. M. Marino[1,2], Luiz G. Gawryszewski[3], and Lucia Riggio[1]*

1. Dipartimento di Neuroscienze, Università di Parma, Parma, Italy

2. Dipartimento di Psicologia, Università di Milano-Bicocca, Milano, Italy
3. Neuroscience Program, Universidade Federal Fluminense, Niterói, Rio de Janeiro, Brazil

* Corresponding author:
Lucia Riggio
Dipartimento di Neuroscienze
Sezione di Fisiologia
Universita` di Parma, Italy.
E-mail address: riggio@unipr.it

## 1. Supplementary Data

Following the Vincentization procedure introduced by Ratcliff (Ratcliff, 1979), we divided the RT distributions for each participant of Experiment 1 (intact handle condition), and for the two levels of the variables Orientation (up vs. down) and Correspondence (corresponding vs. non-corresponding), into 4 quantiles (bins), and we computed mean RTs for each quantile. Then, an ANOVA on RTs with Bins, Orientation and Correspondence as within-subjects factors has been carried out. Besides the main effect of Bins ($F_{3,33} = 433.1$; $p < 0.001$ ; ɳ2 = 0.9) and Correspondence ($F_{1,11} = 42.7$; $p < 0.001$ .064; ɳ2 = 0.68), we found that the interaction between Bins and Correspondence was significant ($F_{3,33} = 5.41$; $p < 0.001$ .064; ɳ2 = 0.17), showing the typical trend of the AE ( Bin 1= 19 ms, SE= 1.5; Bin 2= 24 ms, SE= 0.7; Bin 3=33 ms, SE= 0.7; Bin 4=43 ms SE= 0.5; see supplementary fig 1).

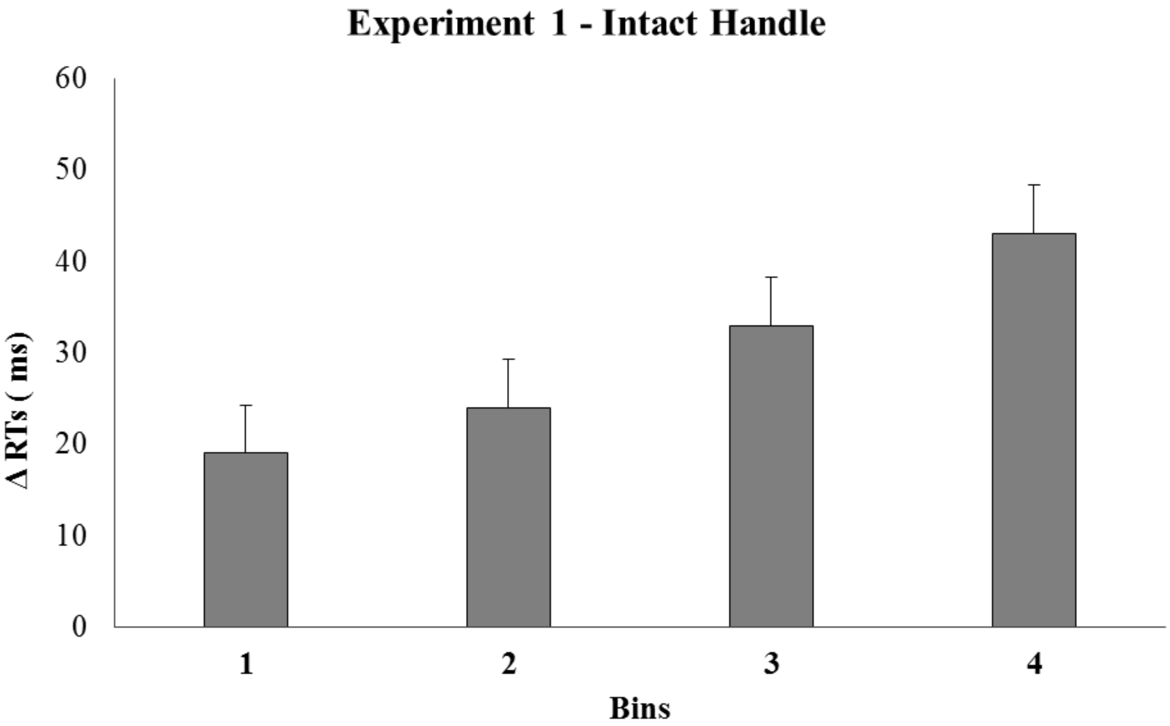


**Supplementary Figure 1.** Δ RTs in Experiment 1 (intact handle condition) as a function of the four bins. The figure shows that magnitude of the AE improves over time. Error bars depict standard errors of the means.

In addition, applying the same Vincentization procedure on the 30 min condition of Experiment 2, we compared it with the intact handle condition of Experiment 1, carrying out an ANOVA having Bins, Orientation and Correspondence as within-subjects factors, and Experiment (previous practice vs. no previous practice) as a between-subjects factor. The ANOVA evidenced in this case too the main effects of Bins ($F_{3,72}$ 1 = 869.3; p <0.001; ɳ2 = 0.9) and Correspondence ($F_{1,24}$ = 68.3; p <0.001 .64; ɳ2 = 0.7), and a significant interaction between Bins and Correspondence ($F_{3,72}$ = 4.3; p <0.01 .; ɳ2 = 0.2). Both the interaction between Experiment and Bins ($F_{3,72}$ = 1.7; p >0.1 .064; ɳ2 = 0.03) and among Experiment, Bins and Correspondence ($F_{3,72}$ = 0.6; p >0.5 .064; ɳ2 = 0.02) were not significant showing the same trend of the AE in the two conditions.
